# Literary Note

**Published:** 1905-07

**Authors:** 


### LITERARY NOTE.

Messrs. Lea Brothers and Company have pleasure in announcing a new edition of Gray's Anatomy, to be published about midsummer, and embodying nearly two years of labor on the part of the editor, Dr. J. Chalmers DaCosta, of Philadelphia, and a corps of special assistants.

The new edition will present a thorough revision, many additions and more than 400 new engravings in black and colors, prepared expressly for this work. In our advertising columns the publishers set forth some of these improvements and we expect later to publish a review of the book.

